# AMEsobreRuedas Early Powered Mobility in Children with Spinal Muscular Atrophy Type I: Protocol of a Randomized Controlled Trial †

**DOI:** 10.3390/jcm13164875

**Published:** 2024-08-18

**Authors:** Rocío Palomo-Carrión, Purificación López-Muñoz, Egmar Longo, Helena Romay-Barrero, Maribel Ródenas-Martínez, María Plasencia-Robledo, Beatriz de Andrés-Beltrán, María Coello-Villalón, Cristina Díaz-López, Soraya Pacheco-da-Costa

**Affiliations:** 1 Department of Nursing, Physiotherapy and Occupational Therapy, Faculty of Physiotherapy and Nursing, Universidad de Castilla-La Mancha, Avda. Carlos III, s/n, 45071 Toledo, Spain; rocio.palomo@uclm.es (R.P.-C.); helena.romay@uclm.es (H.R.-B.); 2 Department of Physical Therapy, Federal University of Paraiba, João Pessoa 58051-900, Brazil; egmarlongo@yahoo.es; 3 Centro de Desarrollo Infantil y Atención Temprana, Asociación APSA, 03005 Alicante, Spain; maribelrm@asociacionapsa.com; 4 Centro Atención Temprana El Grao, Hermanas Hospitalarias, 46024 Valencia, Spain; mplasencia.valencia@hospitalarias.es; 5 Department of Physical Therapy, Centro RIE (Rehabilitación Infantil Especializada), 28050 Madrid, Spain; bdeandres@centrorie.com; 6 Hemichild-Research-Unit, Faculty of Physiotherapy and Nursing, Universidad de Castilla-La Mancha, 45071 Toledo, Spain; maria.coello@uclm.es; 7 Asociación de Padres de Niños con Dificultades en el Desarrollo—APANDID, 45500 Toledo, Spain; cristinadiazlopez0@gmail.com; 8 Neuromusculoskeletal Physical Therapy in Stages of Life Research Group (FINEMEV), Physical Therapy Degree, Department of Nursing and Physical Therapy, Faculty of Medicine and Health Sciences, Universidad de Alcalá, Autovía A2, km 33.200, Alcalá de Henares, 28805 Madrid, Spain; soraya.pacheco@uah.es

**Keywords:** spinal muscular atrophy, power mobility, early intervention, participation, quality of life, family-centered intervention, functional goals

## Abstract

**Background**: Young children with spinal muscular atrophy type 1 (SMA1) have limited independent mobility and participation that may lead to cognitive development delays. Implementing early powered mobility in interventions may help them to learn self-initiated movement, play, and having fun to participate in natural settings. The aim of this study is to evaluate the effectiveness of an early power mobility intervention for increasing participation, functional ability, and quality of life in young children with SMA1. **Methods**: AMEsobreRuedas is a randomized waiting list controlled clinical trial. A sample of 24 children (10 months-5 years old, with SMA1) will be randomly allocated to two groups. The experimental group will perform a family-centered intervention with powered mobility for 16 weeks in their natural environment: a 12-week-structured program three times a week; and a 4-week follow-up with free use of the powered mobility device. The control group (waiting list) will keep their routine and will receive the same intervention after the experimental group. Five assessments will be carried out at baseline and weeks 4, 8, 12, and 16. Primary outcomes are participation (YC-PEM); functional ability (PEDI-CAT); and quality of life (PedsQL-Neuromuscular module). **Results**: It is expected that this study will provide further knowledge about the positive impact of powered mobility for the analyzed variables. Moreover, family engagement in the intervention and establishment of functional goals may help to add valuable information about real needs in future research. **Conclusions**: Early powered mobility could increase the opportunities for children with SMA1 to learn to move independently and participate in their natural environment.

## 1. Introduction

Spinal muscular atrophy (SMA) is an autosomal recessive genetic disorder due to progressive muscle weakness and atrophy. It has an estimated incidence of 1 in 11,000 live births [1]. Children with SMA exhibit a wide spectrum of symptoms with a severity clinically classified from type 0 to 4 (from highest to lowest severity), based on the highest motor milestone achieved [1,2]. Traditionally, SMA classification has been based on the age of symptom onset. However, advances in therapeutic options—such as nusinersen, zolgensma, and risdiplam—along with the possibility of early administration, have been showing the potential to substantially transform the clinical overview. These treatments can increase the levels of the survival motor neuron (SMN) protein, slowing disease progression and improving outcomes [3,4]. According to the consensus statement for standard care in SMA [5], updated in 2018 [6,7] and revised in 2024 [8], the age of symptom onset remains a crucial factor for newly diagnosed infants and those identified through newborn screening. Since motor function and weakness may vary across the musculoskeletal system in young children with SMA, evaluating developmental and motor function prior to treatment is essential. Additionally, proactive rehabilitation should be emphasized to optimize developmental outcomes [8,9]. 

Children diagnosed with SMA type 1 (SMA1) usually present proximal weakness, hypotonia, areflexia, and feeding difficulties within the first 6 months of age. These symptoms lead to delays in motor milestones and significant challenges in achieving independent sitting or walking [10]. Despite these difficulties, recently, the inclusion of disease-modifying therapies has been demonstrating improvements in survival rates and functional capabilities in children with SMA1 [11].

For young children with severe mobility limitations, such as those with SMA1, there can be considerable restrictions in participation and reduced social interaction with caregivers and peers. These limitations often result from the poor self-initiated mobility and delays in learning how to move and cognitive development [12,13]. Dunaway et al. [14] point out that there is a critical therapeutic window for mobility development, suggesting that strategies focused on self-initiated movement can profoundly impact behavior, self-esteem, and social participation. Ideally, providing independent mobility should occur around between 7 and 12 months, as close as possible to the typical ages for crawling and walking, to help minimize delays in cognitive development and facilitate more effective social and motor development [14]. Powered mobility devices offer a means for self-initiated movement and environmental exploration [15]. It is also associated with benefits that extend beyond mobility, potentially improving social skills [14,15,16], communication, cognitive development, and peer interactions [17,18].

Therefore, children with SMA1 are likely to benefit from the early introduction of powered mobility as a valuable strategy intervention for moving, playing, and engaging with their natural environment [17,19]. This approach represents a significant shift in how we support children with SMA, potentially expanding their world and fostering greater social interaction and independence [19,20,21].

A systematic review conducted by Hospodar et al. [20] evaluated the use of modified ride-on cars (MROCs) for children with mobility limitations. The review found that children as young as 7 months could engage with these toys, which enhanced their activity levels and participation. Additionally, MROCs positively impacted family-related outcomes. MROCs are engaging toys that can encourage interaction between infants with mobility limitations and their peers, thereby enhancing social participation [22,23,24]. Positive effects were observed in terms of enjoyment during powered mobility interventions and improved social skills, including better interaction with peers in playgrounds and in playgroups [17,18,20].

Nevertheless, while powered mobility offers numerous functional and developmental benefits, simply incorporating an MROC into a child’s routine is not sufficient. Children also require opportunities to practice in suitable settings to ensure successful learning and integration [21,25,26]. At present, there is a lack of consensus regarding recommendations for interventions that include early powered mobility in terms of intensity, duration, and implementation for children with SMA1, since the evidence remains unclear [21].

Therefore, the aim of this randomized clinical trial (RCT) is to prove the effectiveness of an early powered mobility intervention for enhancing participation, functional ability, and quality of life in young children diagnosed with SMA1. As secondary aims, the impact of the intervention on the level of learning of powered mobility, functional goals, parental stress, and satisfaction with the intervention and with the use of MROC will also be considered.

## 2. Materials and Methods

A single-blinded waiting list RCT will be conducted. This study adheres to ethical principles outlined in the Declaration of Helsinki [27] and the Spanish Law on Personal Data Protection and Guarantee of Digital Rights [28]. It has been approved by the Ethics Committee of the University Hospital in Toledo (Ref No. 842) and is registered on www.clinicaltrials.org (accessed on 18 October 2022) with the ID: NCT05589987.

### 2.1. Study Sample

Children with SMA1, between 10 months and 5 years old, who have no prior experience with powered mobility interventions or have used a powered mobility device for less than 7 h in the month preceding this study will be included. Exclusion criteria are children with severe visual impairments, a body weight exceeding 25 kg—MROC requirement, those who use a respirator, or those with other health conditions affecting the neuromusculoskeletal system that may interfere with the use of powered mobility [26,29].

Sample size calculation was performed using G*Power version 3.1.9.4. The calculation was based on the following parameters: an alpha (α) level of 0.05, which corresponds to a 5% chance of a type I error (incorrectly rejecting the null hypothesis when it is true); a statistical power (1-β) of 0.80, which means there is an 80% chance of correctly rejecting the null hypothesis if there is a true effect; and a medium effect size of 0.5 due to the lack of previous studies in this population and the use of powered mobility. Based on these parameters, the calculation determined that 26 participants, with 13 participants in each group, would be needed to achieve adequate statistical power for this study. To account for a potential dropout rate of 20%, a total of 32 participants will be recruited (*n* = 16 for each group) to ensure that the final sample size remains sufficient for analysis.

Recruitment will be performed at early care centers in collaboration with the Spanish Spinal Muscular Atrophy Foundation (*FUNDAME: Fundación de Atrofia Muscular Espinal*). An informational session will be held with the families of potential participants to explain the aims and procedures of this study.

Once families have shown their desire to participate and signed the informed consent forms, a member of the research team—not involved with assessments or interventions—will randomly assign the participants to two groups: the experimental group and the control group (waiting list group).

### 2.2. Outcome Variables

Outcome variables will be measured by a blind evaluator, member of the research team. Data will be collected at baseline, during intermediate assessments at weeks 4, 8, and 12 throughout the structured program, and at a follow-up assessment at week 16, depending on each specific variable, described as follows.

*Primary outcomes:* measured at baseline, at weeks 8 and 12—during and after the structured intervention—and at week 16 after the follow-up period.
  - Participation: measured with the Spanish version of the Young Children’s Participation and Environment Measure (YC-PEM) [30]. The YC-PEM provides insights into family members’ perspectives on the participation of children from 0 to 5 years old in various activities at home, preschool, and the community. For each activity type, the frequency of participation is rated on a scale from 0 to 7 (0 = never; 7 = one or more times a day). Participation level is rated on a 5-point scale (1 = little involvement; 5 = very involved), and desired changes in participation are recorded as yes or no. Additionally, the impact of environmental characteristics and resources on participation is rated on a scale from 1 to 3 (1 = usually difficult/usually not; 3 = generally helps/usually yes).
  - Functional ability: assessed using the Pediatric Evaluation of Disability Inventory–Computer Adaptive Test (PEDI-CAT) questionnaire [31]. Parents or caregivers complete the PEDI-CAT through an adaptive computer platform. The PEDI-CAT includes a bank of items across two domains: Mobility (75 items, ranging from basic motor skills to more complex skills, including the use of walking devices) and Social/Cognitive (60 items covering interaction, communication, daily cognition, and self-control).
  - Quality of life: evaluated using the Spanish version of the Pediatric Quality of Life Inventory (PedsQL)-Neuromuscular module [32]. The PedsQL is a parent-reported tool designed to evaluate perceptions of the child’s quality of life. It includes three dimensions: 17 questions on neuromuscular disease, 3 questions on communication, and 5 questions about family resources. Responses range from 0 (never) to 4 (almost always), which are converted into a score from 0 to 100. Scores are interpreted as follows: 100—never; 75—almost never; 50—sometimes; 25—frequently; and 0—almost always.
*Secondary outcomes:*
  - Learning of powered mobility: assessed with the Assessment of Learning Powered Mobility (ALP) [33]. The ALP describes 8 levels of performance (1 = beginner; 8 = expert) in using a powered mobility device, based on five categories: attention level; activity and movement; comprehension of the mobility device; expressions and emotions; and interaction and communication [34]. The ALP levels will be assessed weekly to help guide families on the strategies to use during their sessions and ensure consistent progress.
  - Functional goals: measured with the goal attainment scale (GAS) [35] at baseline and at weeks 4, 8, 12, and 16 assessments. The goals focus on the use of powered mobility in the child’s natural environment and will be developed in collaboration with the child’s family and/or caregivers.
  - Parental stress: assessed with the Spanish version of the Parenting Score Index-Short Form (PSI-SF) [36] at baseline and weeks 8, 12, and 16. The PSI-SF is a 36-item self-reported questionnaire that includes three subscales: parental distress (PD), parent–child dysfunctional interaction (PCDI), and difficult child (DC).
  - Satisfaction with home intervention: scored with the Spanish version of the Client Satisfaction Questionnaire (CSQ-8) [37] at weeks 4, 8, and 12. The CSQ-8 measures overall satisfaction with the received service through 8 items scored on a scale from 1 to 4. The total score ranges from 8 to 32, with higher scores indicating greater satisfaction.
  - Satisfaction with the use of the powered mobility device: assessed with the Spanish version of the Quebec User Evaluation of Satisfaction with assistive Technology Version 2.0 (QUEST 2.0) [38] at week 16 by the end of this study. The QUEST 2.0 is a self-administered instrument designed to assess satisfaction with various assistive technologies and related services. It comprises two subscales for devices and services. Each item is rated from 1 (not satisfied) to 5 (very satisfied). The device subscale contains 8 questions addressing the following dimensions: size, durability, weight, safety, fit, comfort, ease of use, and effectiveness. The services subscale contains 4 questions evaluating the provision of services, professional services, repairs, and follow-up support for the device.

### 2.3. Study Flow

**Figure 1:**
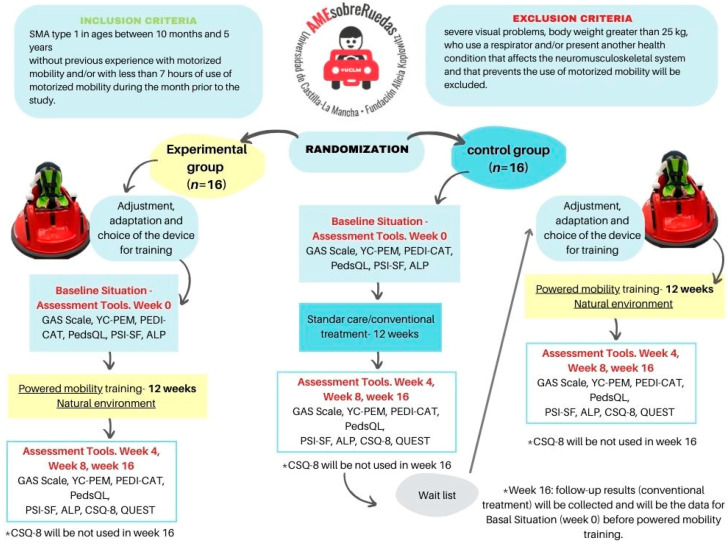
*AMEsobreRuedas* study flowchart.

This study will last 16 weeks. Participants in the experimental group will undergo a structured powered mobility intervention, consisting of 30 min sessions conducted three times a week for 12 weeks. Following this, there will be a 4-week follow-up period during which children in the experimental group have unrestricted use of the powered mobility device. In contrast, participants of the control group will continue with their usual routines during the intervention period. After the 12-week intervention phase, the control group (waiting list) will receive the same powered mobility intervention as the experimental group. Figure 1 illustrates the *AMEsobreRuedas* study flowchart.

### 2.4. Intervention Features

**Figure 2:**
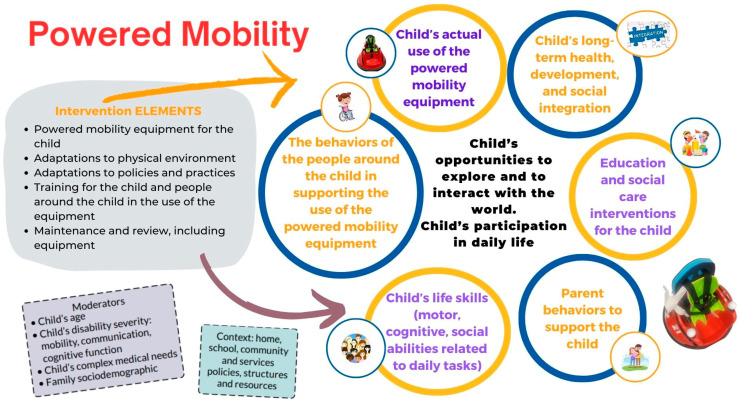
Characteristics of powered mobility intervention for young children [39,40].

It is very important for families to understand both the implementation of the intervention and the operation of the powered mobility device, specifically a modified ride-on car (MROC). Proper training is essential to ensure that children can effectively use the device in their natural environments [39,40]. Figure 2 provides an overview of the key characteristics of interventions involving powered mobility for young children with mobility limitations.

**Figure 3:**
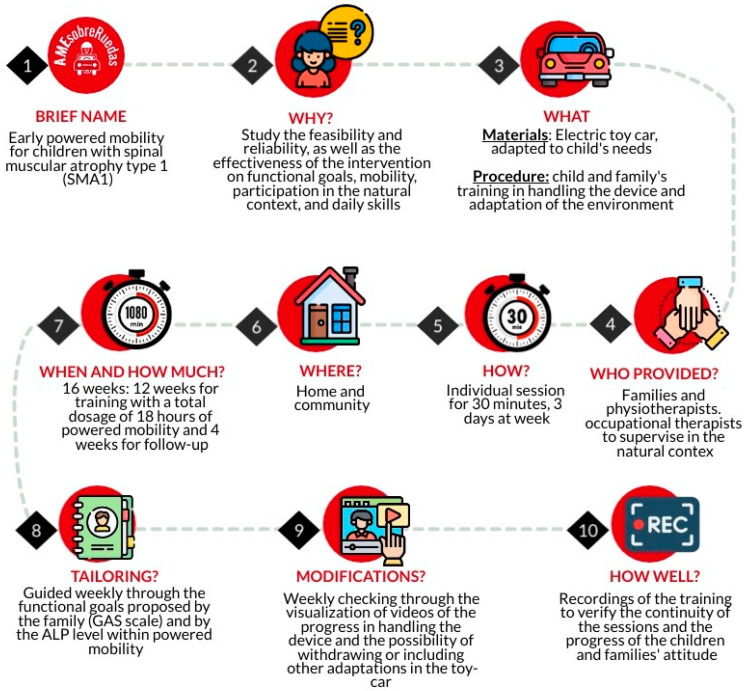
Rehabilitation treatment specification system (RTSS) [41,42,43].

The Rehabilitation Treatment Specification System (RTSS) framework will be used to detail the components of the intervention (Figure 3) [41,42,43]. 

### 2.5. Adaptation of the Powered Mobility Device

**Figure 4:**
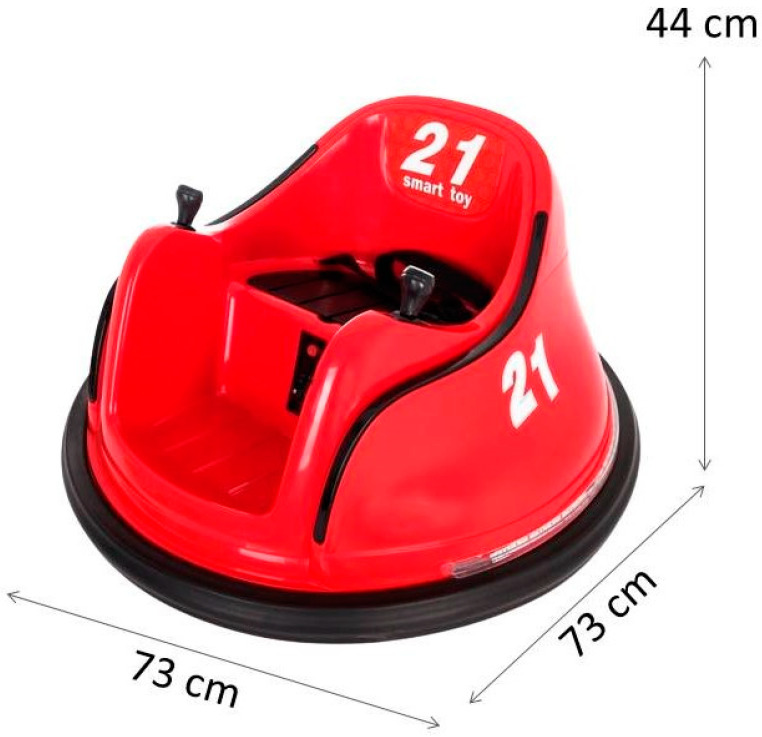
Ride-on-car used for powered mobility intervention in *AMEsobreRuedas*.

Prior to the beginning of the intervention, a face-to-face meeting will be held to individually adjust the powered mobility device that was chosen to be modified (Figure 4). During this meeting, a comprehensive training session will also conducted for the children and their families in order to address the management of the MROC, so they can take it home to use it during the powered mobility intervention. 

**Figure 5:**
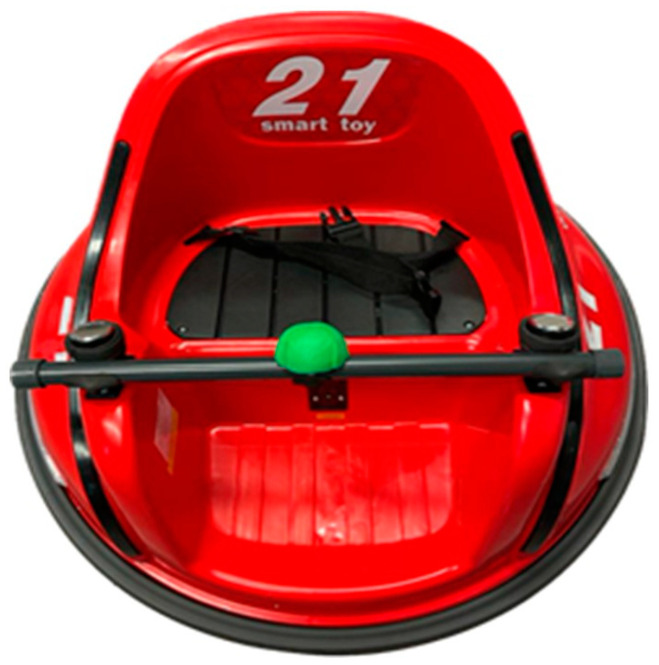
Modified ride-on car featuring a connecting bar that links both levers to assist children with limited grip strength in effectively using their upper extremities.

The modifications and adaptation of the device will be performed based on each participant’s individual needs. They may include the placement of larger wheels, adding protective bumpers, adjusting seats, and customizing controls, among other changes (see Figure 5). These modifications may be either permanent or temporary, depending on the progress towards achieving the specific functional goals set for each child.

### 2.6. Structured Intervention (Weeks 1 to 12) with the Powered Mobility Device

The powered mobility intervention will be family-centered, lasting for 12 weeks, with three 30 min sessions per week in each child’s natural environment, such as home, community, and school settings.

The structured intervention will be guided by an experienced pediatric physical therapist or occupational therapist, who will collaborate in this study, for the implementation of strategies to enhance mobility with the device. Before starting the intervention, the therapists will undergo an 8 h training session conducted by an expert researcher in powered mobility, a member of the research team. This training will cover general powered mobility concepts, how to assess the child’s level using the ALP instrument, and intervention strategies tailored to each ALP level. Additionally, case studies on powered mobility learning will be discussed.

Training sessions will focus on teaching participants about cause and effect through operating the MROCs, such as pressing the joystick to move and releasing it to stop. Families will be instructed on how to adapt the environment to suit the child’s needs and driving conditions, as well as how to use natural play to encourage the child’s driving for environment exploration.

During the structured intervention period, the weekly sessions will be organized as follows:

- **First Weekly Session:** each week, the therapist will conduct the session for approximately one hour. The first 30 min will be dedicated to addressing the family’s needs, evaluating the child’s progress using the ALP, and teaching the family the strategies and structure for the remaining 30 min of training. It is essential for a family member to be present in this session to learn the strategies to be used in the subsequent sessions.
- **Second and Third Weekly Sessions:** these sessions will be conducted by the families and last for 30 min each. They will carry out the training with the MROC, using the strategies they learned during the first session of the week with the therapist.

All sessions will be recorded to allow the therapists and research assessors to review them and then discuss how to optimize the training. Additionally, a member of the research team will conduct weekly follow-up meetings. Families will be required to complete a document detailing the timing of each session, the child’s behavior, family perceptions, and any other relevant observations.

### 2.7. Follow-up Period (Weeks 13 to 16)—Free Use of Powered Mobility Devices

**Figure 6:**
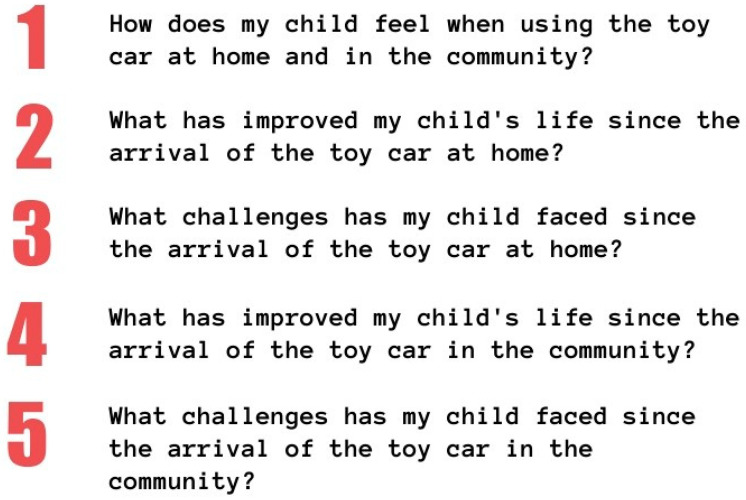
Guiding questions for taking photographs using the photovoice method.

In the final 4 weeks of this study, the children will have unrestricted use of the powered mobility device, allowing them to use it for as long as they want. During this follow-up period, the photovoice method [44] will be employed, guided by participatory action research (PAR) principles [45] and the patient and public involvement approach (PPI) [46,47]. Families will be trained to take high-quality photographs of their children using the device during everyday activities. They will be provided with detailed instructions on how to take the photos, including some guiding questions (see Figure 6), and the dates they have to send the photographs to the research team. Additionally, a script with five guiding questions will be supplied to assist in capturing relevant and informative images. 

**Figure 7:**
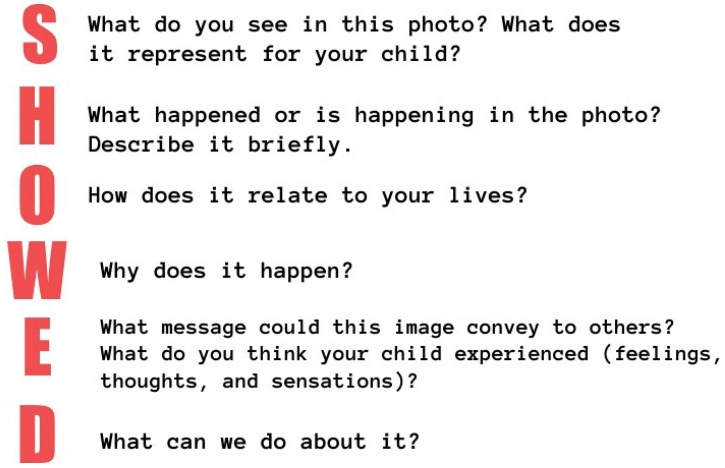
This version clearly describes the purpose and method of the semi-structured script, emphasizing its role in guiding the reflection process.

Each family will be instructed to take at least 4 pictures per week, for a total minimum of 16 over the follow-up period. After the families send all the pictures to the research team, there will be an online discussion group meeting between families and the research team to review the photographs and explore the experiences related to the use of the MROC. An adapted version of the SHOWED methodology [44] will be used (see Figure 7).

**Figure 8:**
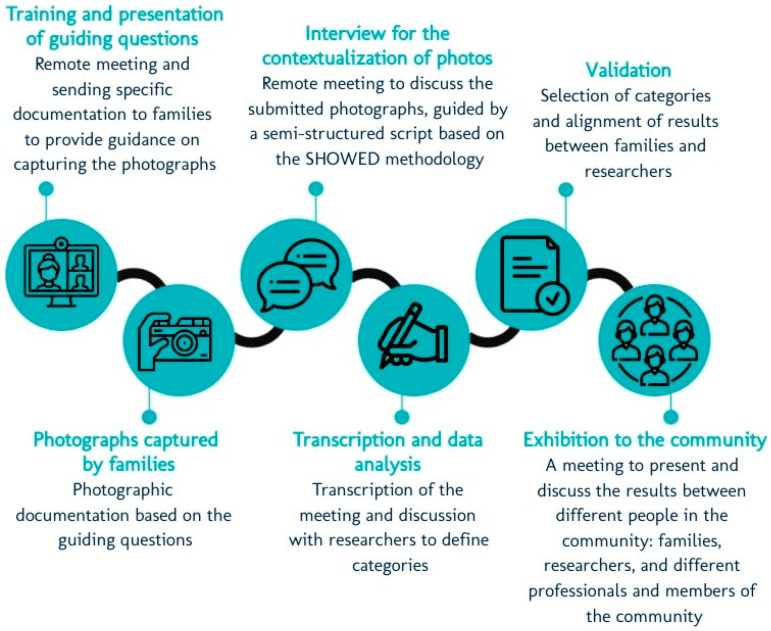
Steps for effective planning and obtaining quality photographic results.

All stages will be conducted remotely using a free app, as illustrated in Figure 8.

### 2.8. Statistical Analysis

Quantitative data will be analyzed using SPSS Statistics 22.00 for Windows. Parametric statistical tests will be employed with a significance level set at *p* < 0.05. To compare outcome variables between interventions, the paired Student’s *t*-test will be employed for comparisons involving two measures. For comparisons involving more than two measures, repeated measures ANOVA will be used to identify specific differences between groups or time points. To provide a comprehensive understanding of the results, effect sizes will be calculated for both the *t*-test and ANOVA. Confidence intervals for these effect sizes will also be reported to offer insights into the robustness of the findings.

Photovoice data will be transcribed by the researchers who conducted the online meetings with the families. The transcriptions will be reviewed by an experienced qualitative researcher [48]. Two researchers will independently analyze the data, followed by a group discussion with the research team to develop preliminary results. Thematic analysis will be performed following the principles outlined by Braun and Clarke [49] with support from ATLAS.ti software v.23. Identified themes will be validated by the participating families, who act as co-investigators in this study. This phase, termed “Validation of Analysis by Parents”, allows families to select the themes that best reflect the content of the sentences and photos included in the analysis.

## 3. Results

This randomized controlled trial (RCT) aims to enhance understanding of the positive impacts of powered mobility on participation, functional ability, and quality of life in children with SMA1. It is anticipated that this intervention will lead to a significant improvement in these areas.

Specifically, it is expected that children will demonstrate an increase of at least 2 points in their ALP level during the structured program. Furthermore, ongoing progress is anticipated throughout the follow-up period, reflecting sustained benefits from the intervention.

Family involvement in the implementation of the intervention and the establishment of functional goals is crucial. Engaging families not only facilitates the practical application of the intervention but also provides valuable insights into the real needs and challenges faced by children with SMA1. This feedback will be instrumental in refining future research and therapeutic strategies.

By exploring these factors, this study aims to offer more precise guidance on optimizing therapeutic approaches for this vulnerable population. The results will provide a more comprehensive overview of the study’s goals and anticipated outcomes, emphasizing the importance of family involvement and the potential impact on future research and therapeutic strategies.

## 4. Discussion

As far as the authors know, this study represents the first RCT focused on early powered mobility for young children with SMA1. The primary aim of this RCT is to evaluate the effectiveness of an early-powered mobility intervention, delivered in the child’s natural environment, for participation, functional ability, and quality of life for young children diagnosed with SMA1 with severe motor impairments. 

There is increasing evidence indicating that early powered mobility training can be highly beneficial for very young children who are unable to move independently and are unlikely to achieve independent mobility in the future [20,22,23,24,50]. In this context, incorporating self-initiated powered mobility through a MROC can play a pivotal role in a comprehensive intervention plan for children with SMA1. 

The MROCs employed in this RCT are electric toys specifically designed to promote self-initiated mobility, enabling children to actively participate in daily activities within their natural environments. By facilitating such mobility, MROCs can enhance children’s independence, autonomy, and self-confidence [25]. Compared to traditional powered wheelchairs, MROCs offer several distinct advantages. They are generally more cost-effective and easier to transport, making them more accessible to a broader range of families. Their toy-like appearance not only makes them more attractive to children but also allows for easier adaptation to the child’s evolving needs and family goals [23,24]. This adaptability is crucial for maintaining engagement and meeting the specific requirements of each child and their family. Despite these advantages, MROCs are not without limitations. They may struggle to navigate uneven terrain or steep slopes, which can restrict their utility in certain environments. Additionally, their design may limit their speed, making them less suitable for rapid outdoor travel. These limitations need to be considered when integrating MROCs into a child’s mobility plan, ensuring that they complement other interventions and address the child’s specific needs effectively. Overall, while MROCs offer a promising and accessible option for enhancing mobility and participation among young children with severe motor impairments, careful consideration of their limitations is essential for optimizing their effectiveness as part of a broader therapeutic strategy.

Evaluating the impact of interventions on participation is crucial, as participation enables children to interact with their peers, acquire knowledge, develop skills, and express creativity, all of which foster cognitive, perceptual, social, and motor development [40]. Participation-based interventions can also improve functional activity and bodily functions, as well as motivation to explore and engage with the environment [16,21]. Therefore, this study may make a significant contribution to evidence-based clinical practice by providing recommendations on the early use of low-cost powered mobility devices in natural settings for children with SMA1.

Family-centered interventions, where parents are actively involved in the training program, can improve the child’s functional performance and empower caregivers by deepening their understanding of their child’s needs. Involving families can also amplify the effects of interventions and assist children in applying learned skills to everyday tasks [51]. Therefore, this study will actively engage families in both the planning and implementation of the intervention, including the formulation of functional goals and the development of the intervention strategy. Families will play a critical role, and children will also be actively involved. During treatment sessions, family members and therapists will encourage children to explore their environment, promote independent mobility, and address mobility challenges encountered during daily activities and social interactions [50,51].

This study may face some limitations. Adherence to the intervention could be challenging due to the fragility and age of the participants. Families of children with SMA1 often have concerns about their children participating in outdoor activities or attending nursery schools. Additionally, the potential for medical complications associated with SMA1 could present challenges. Another potential limitation is the use of the ALP tool, which does not account for the child’s age. Consequently, younger children might not achieve higher levels in the ALP tool, potentially reflecting developmental constraints rather than limitations imposed by the disease.

## 5. Conclusions

Early powered mobility may promote opportunities for children with SMA1 to learn to move independently and participate in their natural environment. The findings will contribute to the development of more effective strategies to enhance mobility, functional outcomes, and overall quality of life for children with SMA1.

## Data Availability

The original contributions presented in this study are included in the article; further inquiries can be directed to the corresponding author.
